# Comparative Analysis of T-Cell Responses to Aquaporin-4 and Myelin Oligodendrocyte Glycoprotein in Inflammatory Demyelinating Central Nervous System Diseases

**DOI:** 10.3389/fimmu.2020.01188

**Published:** 2020-06-17

**Authors:** Livia Sophie Hofer, Melanie Ramberger, Viktoria Gredler, Anna Sophie Pescoller, Kevin Rostásy, Mireia Sospedra, Harald Hegen, Thomas Berger, Andreas Lutterotti, Markus Reindl

**Affiliations:** ^1^Clinical Department of Neurology, Medical University of Innsbruck, Innsbruck, Austria; ^2^Oxford Autoimmune Neurology Group, Nuffield Department of Clinical Neurosciences, University of Oxford, Oxford, United Kingdom; ^3^Paediatric Neurology, Children's Hospital Datteln, Witten/Herdecke University, Datteln, Germany; ^4^Department of Neuroimmunology, University of Zurich, Zurich, Switzerland; ^5^Department of Neurology, Medical University of Vienna, Vienna, Austria

**Keywords:** neuromyelitis optica spectrum disorder, aquaporin-4, myelin oligodendrocyte glycoprotein, antibody, T-cell

## Abstract

Autoantibodies against aquaporin-4 (AQP4-Ab) and myelin oligodendrocyte glycoprotein (MOG-Ab) are associated with rare central nervous system inflammatory demyelinating diseases like neuromyelitis optica spectrum disorders (NMOSD). Previous studies have shown that not only antibodies, but also autoreactive T-cell responses against AQP4 are present in NMOSD. However, no study has yet analyzed the presence of MOG reactive T-cells in patients with MOG antibodies. Therefore, we compared AQP4 and MOG specific peripheral T-cell response in individuals with AQP4-Ab (*n* = 8), MOG-Ab (*n* = 10), multiple sclerosis (MS, *n* = 8), and healthy controls (HC, *n* = 14). Peripheral blood mononuclear cell cultures were stimulated with eight AQP4 and nine MOG peptides selected from previous studies and a tetanus toxoid peptide mix as a positive control. Antigen-specific T-cell responses were assessed using the carboxyfluorescein diacetate succinimidyl ester proliferation assay and the detection of granulocyte macrophage colony-stimulating factor (GM-CSF), interferon (IFN)-ɤ and interleukin (IL)-4, IL-6, and IL-17A in cell culture supernatants. Additionally, human leukocyte antigen (HLA)-DQ and HLA-DR genotyping of all participants was performed. We classified a T-cell response as positive if proliferation (measured by a cell division index ≥3) was confirmed by the secretion of at least one cytokine. Reactivity against AQP4 peptides was observed in many groups, but the T-cell response against AQP4 p156-170 was present only in patients with AQP4-Ab (4/8, 50%) and absent in patients with MOG-Ab, MS and HC (corrected *p* = 0.02). This AQP4 p156-170 peptide specific T-cell response was significantly increased in participants with AQP4-Ab compared to those without [Odds ratio (OR) = 59.00, 95% confidence interval-CI 2.70–1,290.86]. Moreover, T-cell responses against at least one AQP4 peptide were also more frequent in participants with AQP4-Ab (OR = 11.45, 95% CI 1.24–106.05). We did not observe any significant differences for the other AQP4 peptides or any MOG peptide. AQP4-Ab were associated with HLA DQB1^*^02 (OR = 5.71, 95% CI 1.09–30.07), DRB1^*^01 (OR = 9.33, 95% CI 1.50–58.02) and DRB1^*^03 (OR = 6.75, 95% CI = 1.19–38.41). Furthermore, HLA DRB1^*^01 was also associated with the presence of AQP4 p156-170 reactive T-cells (OR = 31.67, 95% CI 1.30–772.98). To summarize, our findings suggest a role of AQP4-specific, but not MOG-specific T-cells, in NMOSD.

## Introduction

Autoantibodies targeting the aquaporin-4 (AQP4) water channel protein and the myelin oligodendrocyte glycoprotein (MOG) are associated with a broad spectrum of human central nervous system (CNS) demyelinating diseases (1, 2). While AQP4-specific antibodies target the AQP4 water channel protein expressed on astrocyte end-feet processes causing a severe astrocytopathy called neuromyelitis optica (NMO) (3, 4), MOG-specific antibodies target the extracellular N-terminal immunoglobulin variable (IgV)-domain of MOG expressed on myelin-forming oligodendrocytes (2, 5, 6). Autoantibodies against AQP4 (AQP4-Ab) have emerged as highly sensitive and specific biomarker for the diagnosis of NMO (3). However, not all patients presenting with clinical features suggestive of an NMO-disease phenotype are positive for AQP4-Ab (7), and a significant proportion of those seronegative patients harbor antibodies to MOG. This created a diagnostic uncertainty reflected in the pathogenetically undefined category of NMO spectrum disorders (NMOSD) proposed in 2015 (1, 2, 8).

Several lines of evidence suggest that autoreactive CD4^+^ T lymphocytes are key players in the pathogenesis of Ab-associated demyelinating CNS diseases. First, passive transfer models using AQP4-specific human IgG are not considered pathogenic without T-cell induced disruption of the blood brain barrier (BBB) (9–11). The exact relevance of T-cell independent pathogenicity of a high affinity rodent monoclonal AQP4-Ab (12) remains to be determined, as serum AQP4-Ab in human NMOSD patients are polyclonal, with a wide range of affinities and often much lower antibody titers (9, 10, 13–15). The serum concentration of AQP4-Ab is many times higher than in the cerebrospinal fluid (CSF) (13, 16–18), and peripheral B cells have the capacity to produce AQP4-Ab *in vitro* (19, 20). Thus, it is supposed that these antibodies are produced outside the CNS and that T effector cells might initiate CNS inflammation leading to BBB disruption and entry of antibodies (6, 9–11, 21–23). Local activation of CD4^+^ T-cells in the CNS is indispensable for providing an inflammatory microenvironment that also enables the initiation of CNS inflammation orchestrating BBB breakdown, lesion location and formation and thus facilitates Ab-mediated disease propagation (6, 11, 23). Second, AQP4-Ab and MOG-Ab are class-switched complement-fixing antibodies depending on T-cell help to be generated emphasizing the pivotal role of antigen-specific T-cell responses. Finally, there is ample evidence that activated T-cells are enriched at lesion sites (11, 24, 25) and that the pathogenic effectors are CD4^+^ T-cells of either T helper (Th)-1 lineage producing pro-inflammatory interferon (IFN)-ɤ or of Th17 lineage producing pro-inflammatory interleukin (IL)-17A (26, 27). Moreover, NMOSD patients also display a higher proportion of Th17 cells or cytokines like IL-6 (28–34).

While the high diagnostic value of AQP4-Ab as hallmark serologic marker in NMOSD has been shown and AQP4-specific T-cells have been examined in NMOSD patients (31, 35–38), the role of MOG-Ab or MOG-specific T-cells is less clear. Since MOG-Ab can be found in up to 50% of AQP4-Ab seronegative NMOSD patients, it is possible that MOG-specific T-cells could play a role in NMOSD development. So far there is no published information about MOG-specific T-cells in NMOSD and related conditions. Until now all studies focused on MOG-specific T-cell responses from MS patients (39–41) or in experimental autoimmune encephalomyelitis (42–44).

Here, we aimed to analyze the T-cell reactivity in response to selected eight AQP4 and nine MOG peptides and their possible restriction to a particular human leukocyte antigen (HLA)-DQ and HLA-DR genotype, and to examine the functional phenotype of autoreactive CD4^+^ T-cells in patients with AQP4-Ab or MOG-Ab.

## Materials and Methods

### Patients and Control Subjects

Eight NMOSD patients with AQP4-Ab, 10 patients with MOG-Ab, 8 patients with MS and 14 healthy controls (HC) were included in this study. NMOSD and MS was diagnosed according to recently published criteria (1, 45, 46). Within the MOG-Ab positive group, one patient also fulfilled the 2015 diagnostic criteria for NMOSD (1), 8 of the other 9 patients had related clinical presentations (three bilateral and one unilateral monophasic optic neuritis, one recurrent optic neuritis, one monophasic and one recurrent myelitis, one acute demyelinating encephalomyelitis with recurrent optic neuritis and one recurrent demyelinating disease) and one patient fulfilled the diagnostic criteria for MS.

Demographic and clinical data of all participants are shown in Table 1.

**Table 1:** CD4^+^ T-cell proliferation with a cell division index ≥ 3 of T-cell cultures after stimulation with AQP4 or MOG peptides.

	**AQP4-Ab^a^**	**MOG-Ab^b^**	**MS**	**HC**	***p*-value^c^**	***p*-value corrected^d^**
Number	8	10	8	14		
Female: male	8: 0	5: 5	7: 1	12: 2		
Age (y)^e^	54 (20–77)	38 (14–53)	47 (24–63)	38 (22–58)		
Duration (y)^e^	4 (0–13)	0 (0–17)	21 (4–34)	n.a.		
Acute relapse	4 (50%)	3 (30%)	0 (0%)	n.a.		
Relapses^e^	4 (1–20)	1 (1–4)	n.a	n.a.		
EDSS^e^	2 (1–7)	1.5 (1–3)	2 (0–7.5)	n.a.		
Treatment	6 (75%)^f^	4 (40%)^g^	6 (75%)^h^	n.a.		
Ab titer	2,560 (40–40,960)	320 (160–10,240)	0 (0–40)	0 (0)		
Tetanus toxoid	8/8 (100%)	10 (100%)	8 (100%)	14 (100%)	0.999	ns
AQP4 p11-30	2/8 (25%)	1/7 (14%)	4/8 (50%)	1/14 (7%)	0.121	ns
AQP4 p61-80	1/8 (13%)	0/7 (0%)	2/8 (25%)	2/14 (14%)	0.570	ns
AQP4 p63-76	2/8 (25%)	0/7 (0%)	2/8 (25%)	3/14 (21%)	0.557	ns
AQP4 p91-110	2/8 (25%)	1/7 (14%)	1/8 (13%)	3/14 (21%)	0.905	ns
AQP4 p139-153	2/8 (25%)	1/7 (14%)	2/8 (25%)	2/14 (14%)	0.878	ns
AQP4 p156-170	4/8 (50)^*^	0/7 (0%)	0/8 (0%)	0/14 (0%)	0.001	0.02
AQP4 p211-230	0/8 (0%)	0/7 (0%)	0/8 (0%)	0/14 (0%)	0.999	ns
AQP4 p281-305	1/8 (13%)	1/7 (14%)	1/8 (13%)	2/14 (14%)	0.999	ns
AQP4 overall	7/8 (88%)	3/7 (43%)	4/8 (50%)	4/14 (29%)	0.066	ns
MOG p1-20	1/5 (20%)	2/10 (20%)	1/8 (13%)	4/14 (29%)	0.846	ns
MOG p35-55	1/5 (20%)	3/10 (30%)	3/8 (38%)	4/14 (29%)	0.926	ns
MOG p64-80	2/5 (40%)	2/10 (20%)	2/8 (25%)	3/14 (21%)	0.841	ns
MOG p81-96	0/5 (0%)	2/10 (20%)	0/8 (0%)	2/14 (14%)	0.453	ns
MOG p99-107	0/5 (0%)	0/10 (0%)	1/8 (13%)	4/14 (29%)	0.167	ns
MOG p119-130	0/5 (0%)	0/10 (0%)	1/8 (13%)	2/14 (14%)	0.523	ns
MOG p181-195	0/5 (0%)	0/10 (0%)	2/8 (25%)	2/14 (14%)	0.300	ns
MOG p186-200	1/5 (20%)	0/10 (0%)	1/8 (13%)	0/14 (0%)	0.237	ns
MOG p205-214	0/5 (0%)	1/10 (10%)	3/8 (38%)	5/14 (36%)	0.216	ns
MOG overall	2/5 (40%)	4/10 (40%)	5/8 (63%)	7/14 (50%)	0.834	ns

a *AQP4-Ab positive patients fulfilling the current diagnostic criteria for neuromyelitis optica spectrum disorders (NMOSD)*.

b *Patients with MOG-Ab (one NMOSD, three bilateral and one unilateral monophasic optic neuritis, one recurrent optic neuritis, one monophasic and one recurrent myelitis, one acute demyelinating encephalomyelitis with recurrent optic neuritis, one recurrent demyelinating disease and one MS)*.

c *Significance of group differences was analyzed using the Chi-square test*,

* *significant difference to HC group*.

d *P-values were adjusted for 20 comparisons using Bonferroni's correction for multiple comparisons*.

e *Data are shown as median (range)*.

f *Immunomodulatory or immunosuppressive treatment with rituximab (2) or azathioprine (2)*.

g *Immunomodulatory or immunosuppressive treatment with rituximab (2) or plasma exchange (2)*.

h *Immunomodulatory or immunosuppressive treatment with rituximab (1), interferon-β (1), natalizumab (2), azathioprine (1) and teriflunomide (1). Due to limited sample availability not all AQP4-Ab or MOG-Ab positive patients were investigated for T-cell reactivity against AQP4 or MOG peptides. Ab, antibody; AQP4, aquaporin-4; CDI, cell division index; CFSE, carboxyfluorescein succinimidyl ester; HC, healthy controls; MOG, myelin oligodendrocyte glycoprotein; MS, multiple sclerosis; n.a., not applicable/available; ns, statistically not significant after correction for multiple comparisons*.

All samples were collected between 2008 and 2018 at the Clinical Department of Neurology Innsbruck and at the Section for Neuroimmunology and MS Research (NIMS), Department of Neurology, University Hospital Zurich and stored at the Neurological Research Laboratory Innsbruck.

The study was approved by the local Ethics Committee of Medical University of Innsbruck, Austria (study number AN3041) and University of Zürich, Switzerland (KEK ZH 2013-0001) and all patients or their caregivers and controls gave written informed consent.

### AQP4-Ab and MOG-Ab Detection Assays

Serum AQP4-Ab were analyzed using live cell-based immunofluorescence assays as described previously (47). Serum MOG-Ab were analyzed using recombinant live cell-based immunofluorescence assays with HEK293A cells transfected with full-length MOG (human MOG α-1 EGFP fusion protein) as described previously (47). Sera were tested at dilutions of 1:20 and 1:40 and MOG-Ab positivity was titrated with serial dilutions with a threshold of 1:160 to define MOG-Ab positivity. Isolated IgM reactivity was excluded using IgG constant chain (Fc)-specific secondary antibodies (48, 49).

### T-Cell Epitope Mapping Using the CFSE Proliferation Assay

**Figure 1:**
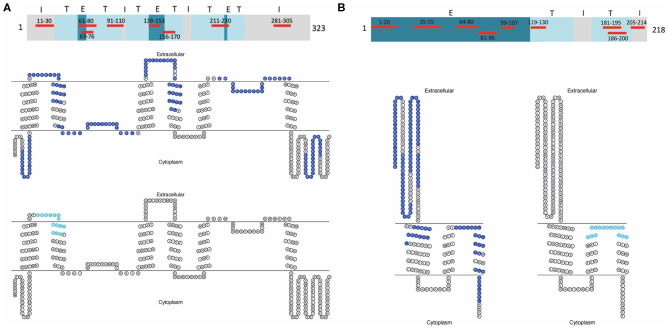
AQP4 and MOG peptides used for CD4^+^ T-cell stimulation. **(A)** Topological map of the human AQP4 protein (323 aa). Eight selected AQP4 peptides corresponding to intracellular (I), extracellular (E), and transmembrane (T) sequences of human AQP4 used for CD4^+^ T-cell stimulation are highlighted in red. Below, seven AQP4 determinants (blue) and further down one overlapping AQP4 determinant (aqua) are represented within a human AQP4 topological diagram using TOPO2 transmembrane protein display software (http://www.sacs.ucsf.edu/TOPO2/). **(B)** Topological map of the human MOG protein (218 aa). Nine selected MOG peptides corresponding to intracellular (I), extracellular (E), and transmembrane (T) sequences of human MOG used for CD4^+^ T-cell stimulation are highlighted in red. Below on the left, eight MOG determinants (blue) and on the right, one overlapping MOG determinant (aqua) are represented within a human MOG topological diagram using TOPO2 transmembrane protein display software (http://www.sacs.ucsf.edu/TOPO2/). AQP4, aquaporin-4; MOG, myelin oligodendrocyte glycoprotein.

**Figure 2:**
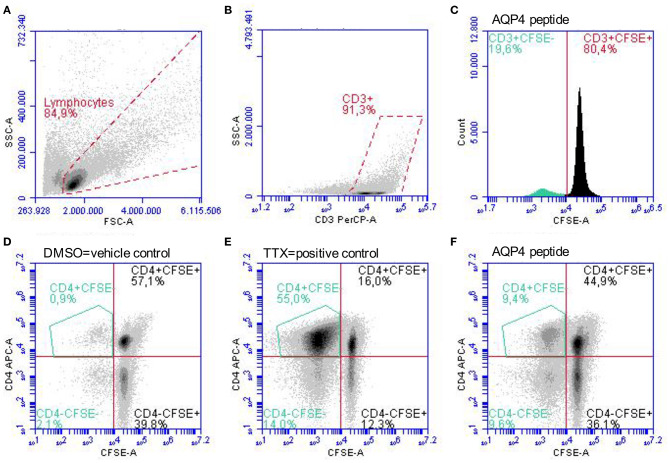
Gating strategy for the identification of proliferated CD4^+^CFSE^−^ T-cells. PBMC stimulated with single AQP4 and MOG peptides or the vehicle control DMSO and the positive control TTX were analyzed after 11 days in culture. **(A)** Gating of lymphocytes according to empirical values of size (FSC) and granularity (SSC) followed by **(B)** gating of CD3^+^ T-cells is shown. **(C)** Dilution of the CFSE staining due to proliferating of CD4^+^ T-cells in response to an AQP4 peptide. **(D–F)** Gating of proliferated CD4^+^CFSE^−^ T-cells. Representative scatter plots of proliferated CD4^+^CFSE^−^ T-cells in response to the vehicle control DMSO **(D)**, to the positive control TTX **(E)**, and to an AQP4 peptide **(F)** are depicted. APC, allophycocyanin; AQP4, aquaporin-4; CFSE, carboxyfluorescein succinimidyl ester; DMSO, dimethyl sulfoxide; FSC, forward scatter; MOG, myelin oligodendrocyte glycoprotein; PBMC, peripheral blood mononuclear cells; PerCP, peridinin-chlorophyll-protein; SSC, side scatter; TTX, tetanus toxoid.

Peripheral blood mononuclear cells (PBMC) were isolated by density gradient centrifugation over Histopaque 1077 (Sigma-Aldrich, St. Louis, MO, USA) according to the manufacturer's instructions and aliquots at a concentration of 1–2 × 10^7^ cells/ml freezing medium (50% Roswell Park Memorial Institute (RPMI) 1,640 medium, 40% fetal calf serum (FCS), 10% dimethyl sulfoxide (DMSO; Sigma-Aldrich, St. Louis, MO, USA) were cryopreserved in liquid nitrogen until use. After thawing adopting a warm and slow processing method as recommended previously (50) to ensure high viability, isolated PBMC of patients and controls at a concentration of 2 × 10^7^ cells/ml were stained with 0.4 μM carboxyfluorescein diacetate succinimidyl ester (CFSE; Life Technologies, Carlsbad, CA, USA) following the manufacturer's instructions and cells were cultivated in *X-Vivo* 15 growth medium (Lonza, Basel, Switzerland). For the expansion of antigen-specific T-cells, PBMC were exposed to 20 μg/ml of selected AQP4 and MOG peptides. Eight AQP4 and nine MOG peptides were selected based on their encephalitogenicity in animals and/or their immunodominance in humans, in particular AQP4-specific T-cell responses of PBMC from NMOSD patients and MOG-specific T-cell responses of PBMC from MS patients (Figure 1, Table 2; (21, 22, 31, 35–41, 43, 51–56). Peptide lengths varied from 9 to 25 amino acids (aa) and were synthesized by Peptides & Elephants (Potsdam, Germany). As positive control, 5 μg/ml tetanus toxoid pool (TTX; Peptides&Elephants, Potsdam, Germany) and as vehicle control, DMSO (Sigma-Aldrich, St. Louis, MO, USA) was used. Since DMSO was used to dissolve the peptides at a maximum of 35% (v/v) in Dulbecco's phosphate-buffered saline (DPBS; Sigma-Aldrich, St. Louis, MO, USA), a 35% DMSO/65% DPBS mix was added at an equal volume to match the volume of added peptide solution. Moreover, a second positive control with the strong mitogen phytohaemagglutinin (PHA; Sigma-Aldrich, St. Louis, MO, USA) was included. Wells from PHA-stimulated cells were evaluated on day 4 for color change of the medium (from red to yellow) indicative of a high metabolic and proliferative activity and by dilution of the CFSE staining using flow cytometry. Cells were seeded at a density of 2 x 10^5^ cells/200 μl in tissue culture test plates 96 U (TPP, Trasadingen, Switzerland), each six wells per condition. After eight days, cells were re-stimulated with half the amount of respective peptides (10 μg/ml per peptide) or vehicle and positive control and 100 μl of the supernatant were replaced with fresh medium containing 20 U/ml IL-2 (Peprotech, Hamburg, Germany), and supernatants were stored at −80°C for later cytokine analyses. After a further 3 days, PBMC were harvested and the proliferation of CD4^+^ T-cells in response to single peptides was analyzed via the dilution of the CFSE staining using flow cytometry. For flow cytometry analysis, PBMC were stained with Peridinin-Chlorophyll-Protein (PerCp)-anti-CD3 (SK7), phycoerythrin (PE)-anti-CD8 (SK1) and allophycocyanin (APC)-anti-CD4 (SK3) antibodies (all BD Bioscience, Franklin Lakes, NJ, USA) and analyzed on an Accuri C6 flow cytometer (BD Bioscience, Franklin Lakes, NJ, USA). The gating strategy is shown in Figure 2. For analysis of a positive T-cell proliferative response, the cell division index (CDI) was calculated as follows, whereby a CDI ≥ 3 was considered as significant proliferation:

$$\begin{array}{l} \text{CDI }:\;\frac{CD4^{+}CFSE^{-}\;cells\;stimulated\;with\;either\;AQP4\;or\;MOG\;peptides\;or\;TTX\;\left(\%\right)}{vehicle-treated\;CD4^{+}CFSE^{-}\;cells\;\left(\%\right)} \end{array}$$

**Table 2:** AQP4 and MOG peptides used for CD4^+^ T-cell stimulation.

**Peptide**	**Sequence**	**aa**	**Mr**	**References**
**AQP4 peptides**				
11–30	GKCGPLCTRENIMVAFKGVW	20	2,209	(31, 36)
61–80	GTEKPLPVDMVLISLCFGLS	20	2,119	(31, 36)
63–76	EKPLPVDMVLISLC	14	1,556	(31)
91–110	ISGGHINPAVTVAMVCTRKI	20	2,067	(36)
139–153	PSVVGGLGVTMVHGN	15	1,423	(31, 51)
156–170	AGHGLLVELIITFQL	15	1,623	(31)
211–230	SMNPARSFGPAVIMGNWENH	20	2,215	(31, 36)
281–305	EDNRSQVETDDLILKPGVVHVIDVD	25	2,805	(37)
**MOG peptides**				
1–20	GQFRVIGPRHPIRALVGDEV	20	2,216	(39, 52)
35–55	MEVGWYRPPFSRVVHLYRNGK	21	2,591	(39, 42, 52)
64–80	EYRGRTELLKDAIGEGK	17	1,934	(52)
81–96	VTLRIRNVRFSDEGGF	16	1,865	(52)
99–107	FFRDHSYQE	9	1,227	(53)
119–130	FYWVSPGVLVLL	12	1,392	(41, 43)
181–195	TLFVIVPVLGPLVAL	15	1,550	(41)
186–200	VPVLGPLVALIICYN	15	1,583	(41)
205–214	RLAGQFLEEL	19	1,174	(54)

*Eight AQP4 and nine MOG peptides corresponding to intra-, extracellular and transmembrane domains of human AQP4 and MOG were selected based on their encephalitogenicity in animals and/or their immunodominance in humans*.

*Aa, number of amino acids; AQP4, aquaporin-4; MOG, myelin oligodendrocyte glycoprotein; Mr, molecular weight*.

### Evaluation of Cytokine Secretion Using ELISA

For the evaluation of cytokine secretion of autoreactive T-cells in response to either AQP4 or MOG peptides, commercial ELISA kits specific for human granulocyte-macrophage-colony-stimulating factor (GM-CSF) and IFN-ɤ (BioLegend; San Diego, USA) and for human IL-4, IL-6, and IL-17A (Thermo Fischer Scientific, Waltham, MA, USA) were purchased and cell culture supernatants collected after 11 days (72 h after re-stimulation) were analyzed following the manufacturer's instructions. The stimulation index (SI) was calculated as follows, whereby a SI ≥ 3 was considered as significant secretion:

$$\begin{array}{l} \text{SI }:\;\frac{secreted\;cytokines\;of\;PBMC\;stimulated\;with\;either\;AQP4\;or\;MOG\;peptides\;or\;TTX\;\left(pg/ml\right)}{secreted\;cytokines\;of\;vehicle-treated\;PBMC\;\left(pg/ml\right)} \end{array}$$

### HLA Typing by Sequence-Specific Primers (PCR-SSP-HLA Typing)

Since binding of peptides to major histocompatibility complex (MHC) molecules of antigen presenting cells (APC) is an important prerequisite for T-cell responsiveness, an HLA-DQ und -DR type determination was performed using polymerase chain reaction with sequence-specific primer (PCR-SSP) technique according to the manufacturer's instructions (Olerup SSP, Stockholm, Sweden).

### Statistical Analysis

The primary hypothesis of this study was that T-cell responses are associated with auto-antibody responses, i.e., AQP4-specific T-cells are increased in participants with AQP4-Ab and MOG-specific T-cells are increased in participants with MOG-Ab. This hypothesis was tested for nominal data (i.e., proliferation with a CDI ≥ 3) using the Chi-square test (with Fisher's exact test and Bonferroni's correction for multiple comparison for subgroups). Statistical significance was defined as two-sided *p* < 0.05 after Bonferroni's correction for multiple comparisons (i.e., the number of different peptides used). According to recently published recommendations to avoid the overuse and misinterpretation of *p*-values, the analysis of all secondary and other endpoints focused on estimates (common odds ratio, OR) and 95% confidence intervals (CI) (57). Statistical analyses were performed using IBM SPSS software (IBM SPSS Statistics; Version 24.0. Armonk, NY: IBM Corp.), GraphPad Prism 8 (GraphPad Software, La Jolla, CA) and OpenMetaAnalyst (http://www.cebm.brown.edu/openmeta/).

## Results

### AQP4-Ab Are Associated With AQP4-Specific CD4^+^ T-Cell Reactivity

**Figure 3:**
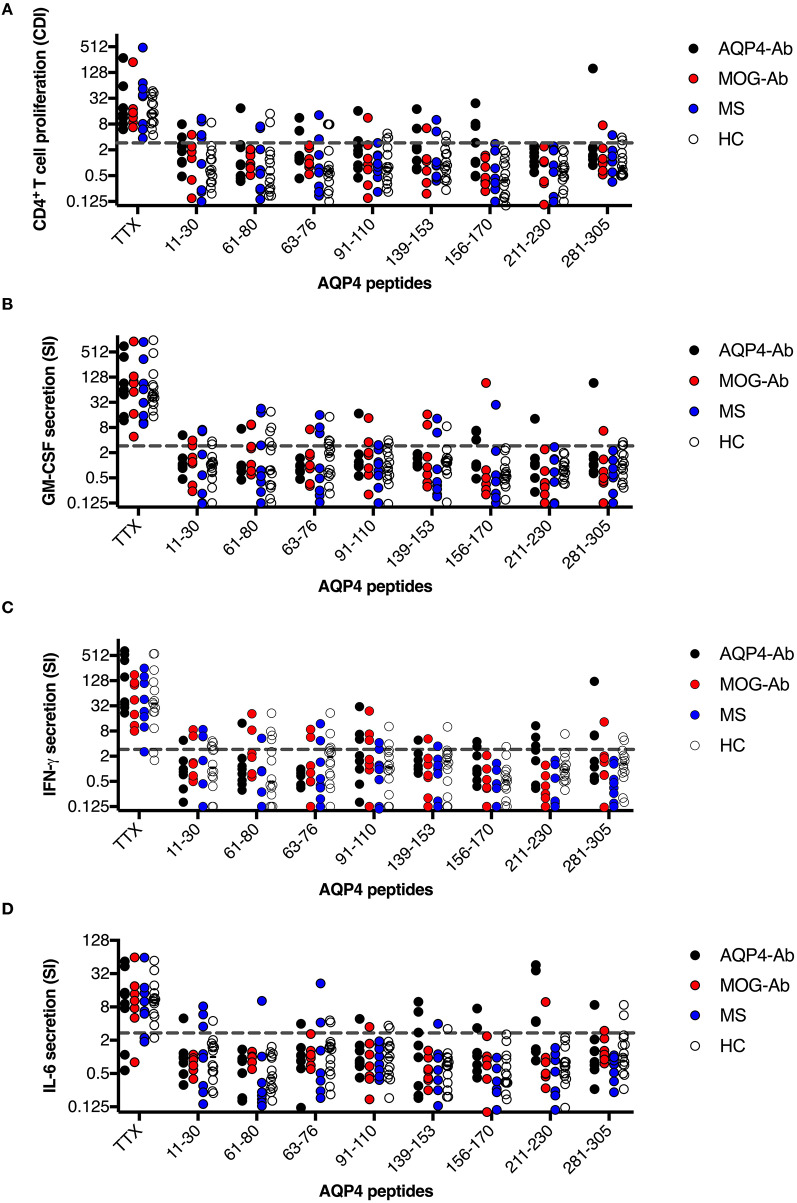
CD4^+^ T-cell reactivity to AQP4 peptides in participants with AQP4-Ab (*n* = 8), MOG-Ab (*n* = 7), MS (*n* = 8) and HC (*n* = 14). **(A)** CD4^+^ T-cell proliferation after challenging with respective AQP4 peptides and the positive control TTX. The cut-off value of a CDI ≥ 3 is indicated by a gray dashed line. Secretion of GM-CSF **(B)**, IFN-γ **(C)** and IL-6 **(D)** after challenging with respective AQP4 peptides and the positive control TTX. The cut-off SI ≥ 3 is indicated by a gray dashed line. AQP4-Ab, aquaporin-4 antibody positive; CDI, cell division index; GM-CSF, granulocyte-macrophage-colony-stimulating factor; HC, healthy controls; IFN, interferon; IL, interleukin; MOG-Ab, myelin oligodendrocyte glycoprotein antibody positive; MS, multiple sclerosis; SI, stimulation index; TTX, tetanus toxoid.

**Figure 4:**
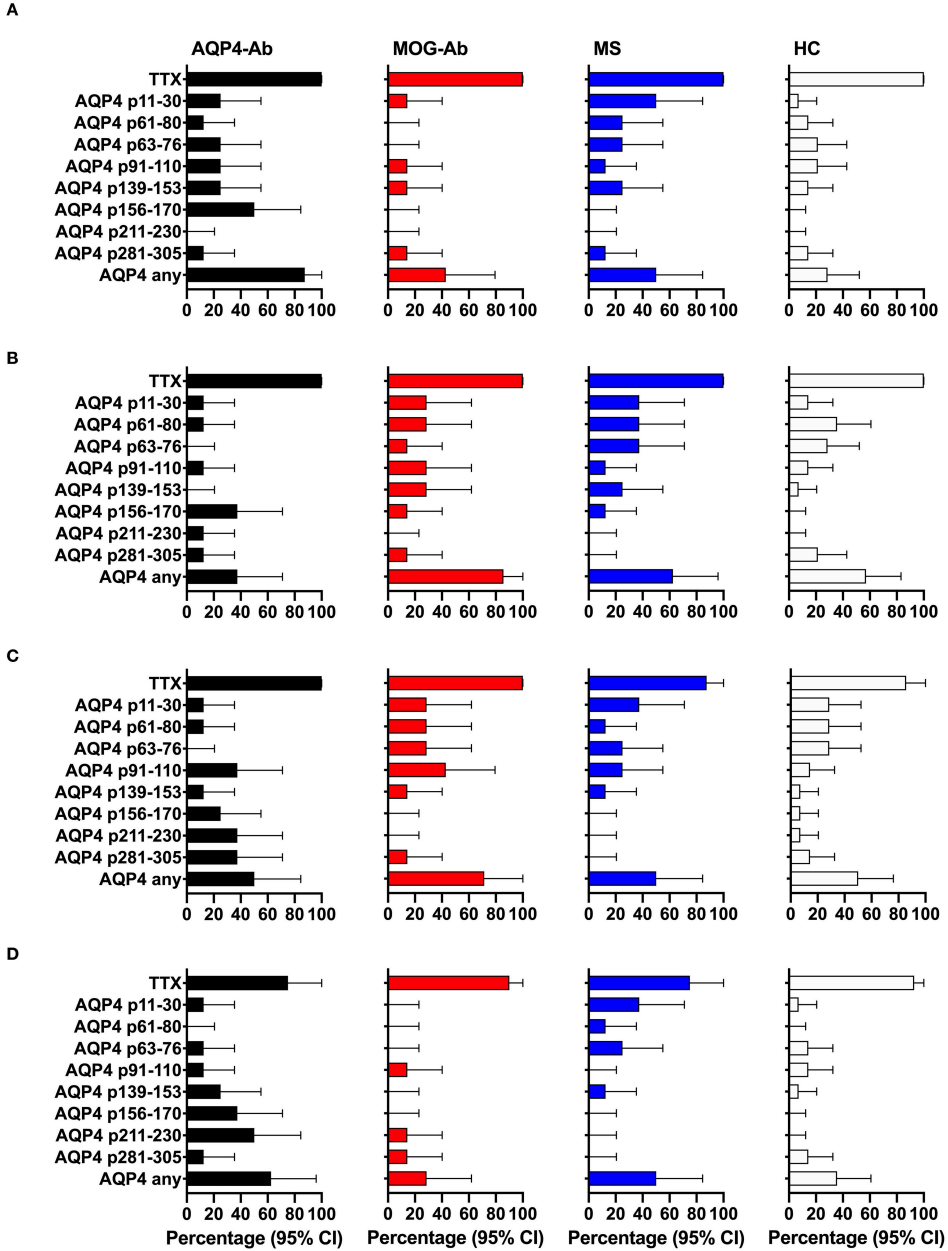
CD4^+^ T-cell reactivity to AQP4 peptides in participants with AQP4-Ab (*n* = 8), MOG-Ab (*n* = 7), MS (*n* = 8) and HC (*n* = 14). **(A)** percentage of participants with positive CD4^+^ T-cell proliferation (CDI ≥ 3) after challenging with respective AQP4 peptides and the positive control TTX. Percentage of participants with positive secretion (SI ≥ 3) of GM-CSF **(B)**, IFN-γ **(C)** and IL-6 **(D)** after challenging with respective AQP4 peptides and the positive control TTX. The 95% confidence intervals are indicated by the error bars. AQP4-Ab, aquaporin-4 antibody positive; CDI, cell division index; GM-CSF, granulocyte-macrophage-colony-stimulating factor; HC, healthy controls; IFN, interferon; IL, interleukin; MOG-Ab, myelin oligodendrocyte glycoprotein antibody positive; MS, multiple sclerosis; SI, stimulation index; TTX, tetanus toxoid.

**Figure 5:**
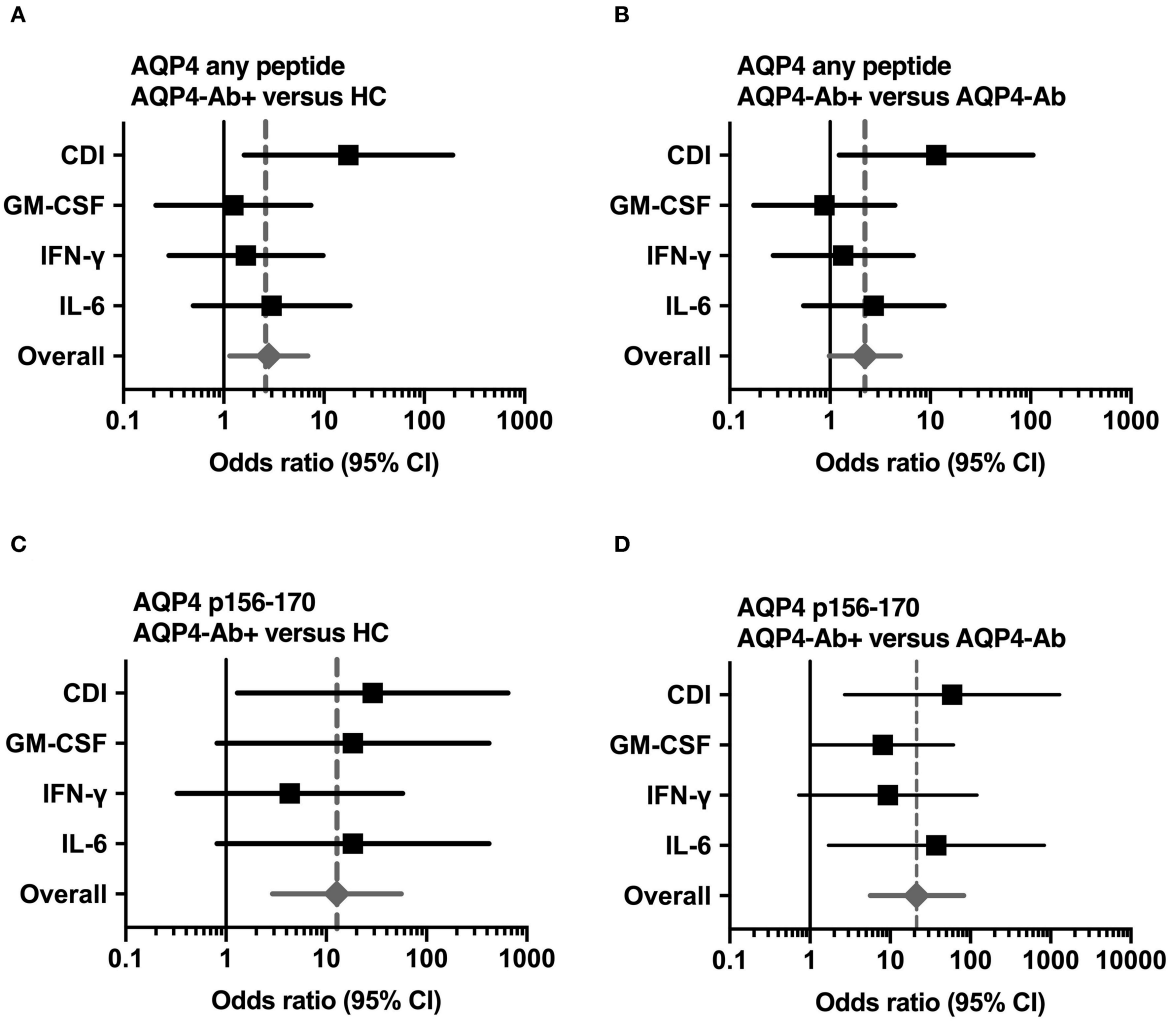
Significant CD4^+^ T-cell reactivity to AQP4 peptides. Forest plot displaying Mantel-Haenszel common odds ratio estimates (symbols) with asymptomatic 95% CI (horizontal lines) for response to AQP4 peptides. **(A)** Reactivity against at least one AQP4 peptide, comparison of patients with AQP4-Ab (*n* = 8) vs. HC (*n* = 14). **(B)** Reactivity against at least one AQP4 peptide, comparison of patients with AQP4-Ab (*n* = 8) vs. all AQP4-Ab negative participants (*n* = 29). **(C)** Reactivity against AQP4 p156-170, comparison of patients with AQP4-Ab (*n* = 8) vs. HC (*n* = 14). **(D)** Reactivity against AQP4 p156-170, comparison of patients with AQP4-Ab (*n* = 8) vs. all AQP4-Ab negative participants (*n* = 29). AQP4-Ab^+^, aquaporin-4 antibody positive; AQP4-Ab-, aquaporin-4 antibody negative, CDI, cell division index; CI, confidence interval; GM-CSF, granulocyte-macrophage-colony-stimulating factor; HC, healthy controls; IFN, interferon; IL, interleukin.

We adopted a cell division analysis procedure based on the quantitative dilution of the fluorescent dye CFSE to investigate the CD4^+^ T-cell autoreactivity of individuals with AQP4-Ab, MOG-Ab, MS and HC against selected AQP4 peptides. All participants showed a positive CD4^+^ T-cell proliferative response with a CDI ≥ 3 to the positive control TTX (Figures 3, 4A and Table 1). T-cell proliferation with a CDI ≥ 3 for at least one AQP4 peptide was observed in the majority of patients with AQP4-Ab (88%), 43% of patients with MOG-Ab, 50% of MS patients and 29% of HC. These proliferative T-cell responses against at least one AQP4 peptide were more frequent in participants with AQP4-Ab as compared to HC (OR = 17.50, 95% CI 1.60–191.89) or all AQP4-Ab negative participants (OR = 11.45, 95% CI 1.24–106.05; Figure 5). Amongst the different AQP4 peptides, a statistically significant response was only seen for AQP4 p156-170: T-cell proliferation with a CDI ≥ 3 was observed in 4/8 (50%) of patients with AQP4-Ab but in none of the other groups (corrected *p*-value 0.02). Proliferative T-cell responses against AQP4 p156-170 were significantly more frequent in participants with AQP4-Ab as compared to HC (OR = 29.00, 95% CI 1.30–648.44) or all AQP4-Ab negative participants (OR = 59.00, 95% CI 2.70–1,290.86; Figure 5).

The clinical presentation of the 4 patients who had increased T-cell reactivity to AQP4 p156-170 were not substantially different from the other AQP4-Ab NMOSD patients. All four cases (all female, age 20–53 years, disease duration 0.4–13.2 years) had a relapsing NMOSD disease course (2–8 relapses), three of them were treated with rituximab and the fourth patient was under high-dose corticosteroids before the initiation of rituximab treatment. Two of the four patients had a relapse at the time of blood sampling, one of them before the initiation of rituximab treatment.

The functional phenotype of proliferating T-cells was characterized by investigating the secretion of the cytokines IL-4, IL-6, IL-17A, GM-CSF, and IFN-ɤ into cell culture supernatants of the CFSE proliferation assay using ELISA. Cytokine concentrations of IL-4 and IL-17A after stimulation with AQP4 peptides, but not after stimulation with TTX, were below the detection limit of ELISA. Quantitative and qualitative values for GM-CSF, IFN-ɤ, or IL-6 levels are shown in Figures 3, 4B–D. A comprehensive analysis of all proliferative and cytokine responses to any AQP4 peptide and p156-170 is shown in Figure 5. From this figure it is evident that overall these responses are increased in AQP4-Ab positive patients as compared to HC or all AQP4-Ab negative participants.

### No Association of MOG-Ab With MOG-Specific CD4^+^ T-Cell Reactivity

**Figure 6:**
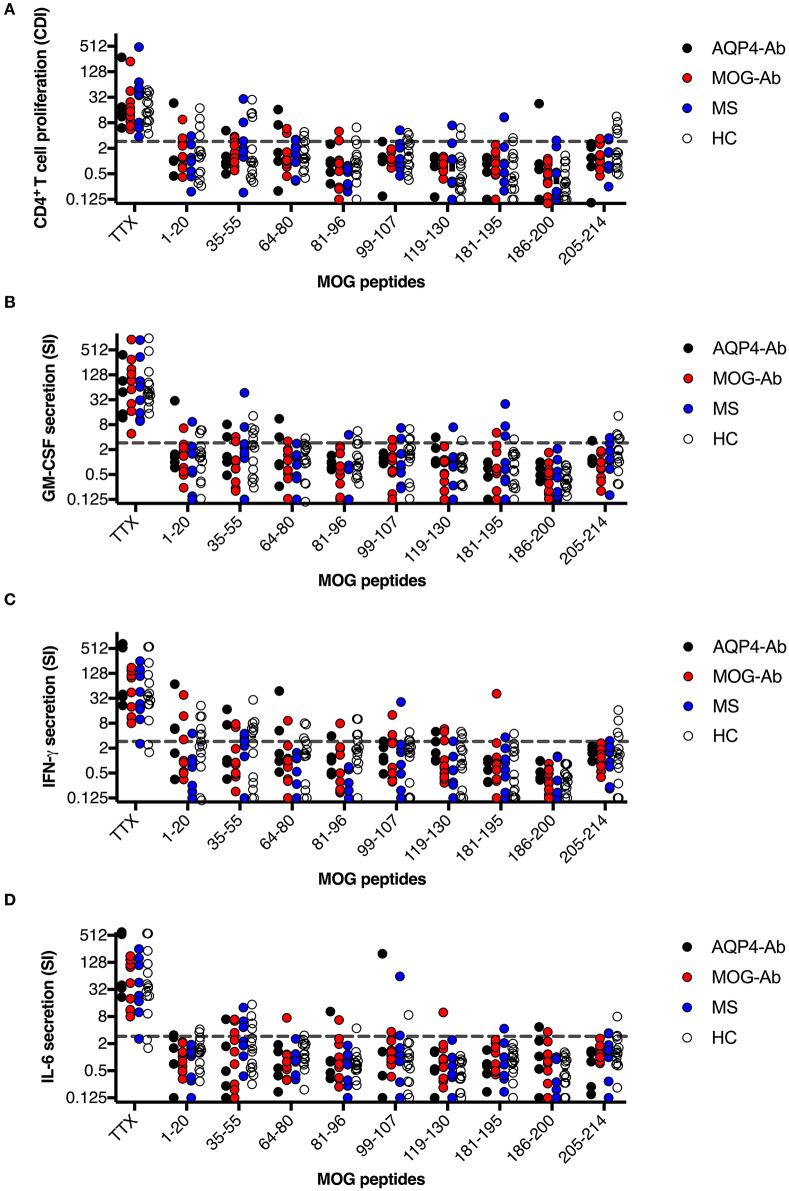
CD4^+^ T-cell reactivity to MOG peptides in participants with AQP4-Ab (*n* = 5), MOG-Ab (*n* = 10), MS (*n* = 8) and HC (*n* = 14). **(A)** CD4^+^ T-cell proliferation after challenging with respective MOG peptides and the positive control TTX. The cut-off value of a CDI ≥ 3 is indicated by a gray dashed line. Secretion of GM-CSF **(B)**, IFN-γ **(C)** and IL-6 **(D)** after challenging with respective MOG peptides and the positive control TTX. The cut-off value of a SI ≥ 3 is indicated by a gray dashed line. AQP4-Ab, aquaporin-4 antibody positive; CDI, cell division index; GM-CSF, granulocyte-macrophage-colony-stimulating factor; HC, healthy controls; IFN, interferon; IL, interleukin; MOG-Ab, myelin oligodendrocyte glycoprotein antibody positive; MS, multiple sclerosis; SI, stimulation index; TTX, tetanus toxoid.

**Figure 7:**
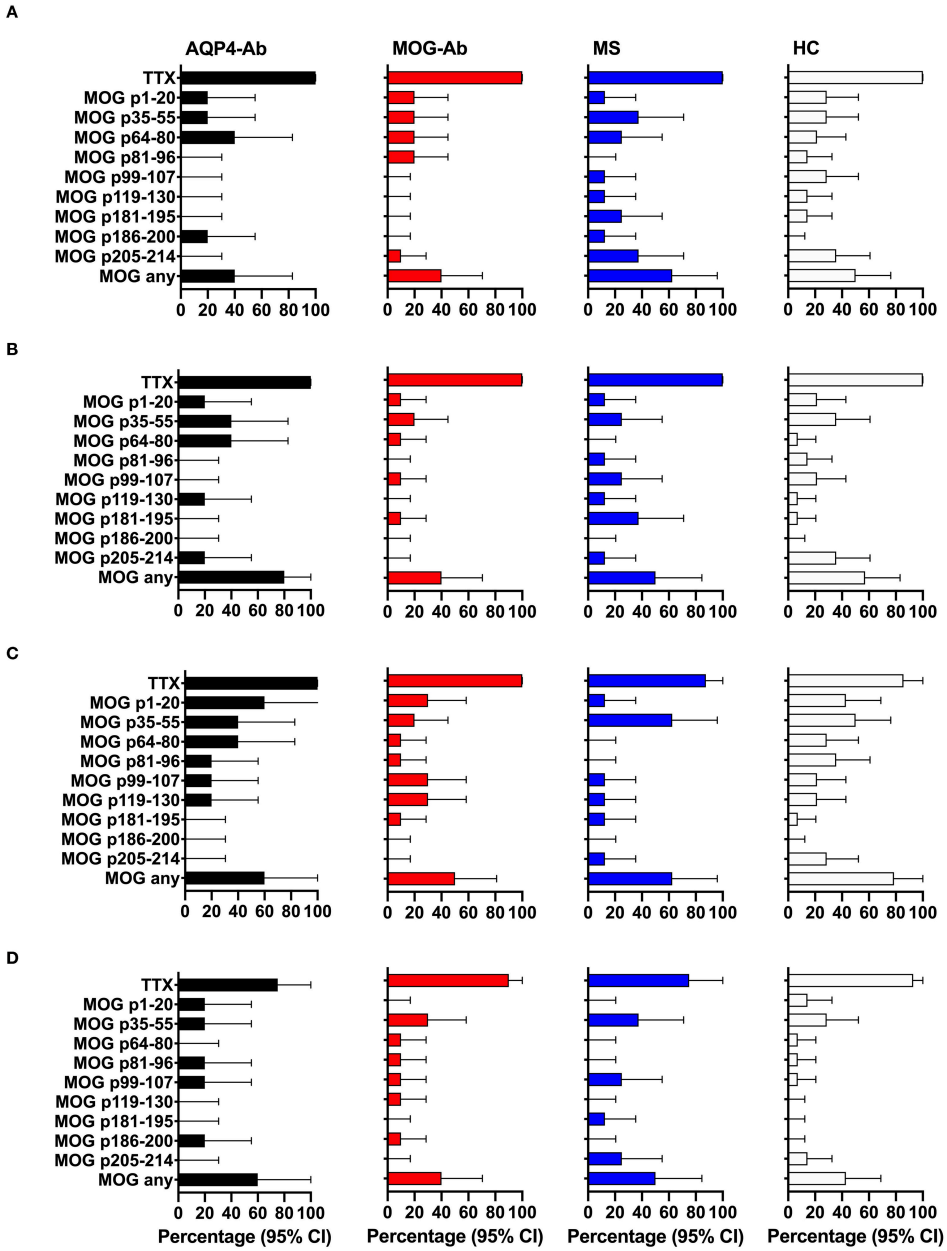
CD4^+^ T-cell reactivity to MOG peptides in participants with AQP4-Ab (*n* = 5), MOG-Ab (*n* = 10), MS (*n* = 8) and HC (*n* = 14). **(A)** Percentage of participants with positive CD4^+^ T-cell proliferation (CDI ≥ 3) after challenging with respective MOG peptides and the positive control TTX. Percentage of participants with positive secretion (SI ≥ 3) of GM-CSF **(B)**, IFN-γ **(C)** and IL-6 **(D)** after challenging with respective MOG peptides and the positive control TTX. The 95% confidence intervals are indicated by the error bars. AQP4-Ab, aquaporin-4 antibody positive; CDI, cell division index; GM-CSF, granulocyte-macrophage-colony-stimulating factor; HC, healthy controls; IFN, interferon; IL, interleukin; MOG-Ab, myelin oligodendrocyte glycoprotein antibody positive; MS, multiple sclerosis; SI, stimulation index; TTX, tetanus toxoid.

In a next step, we analyzed the CD4^+^ T-cell autoreactivity of patients with AQP4-Ab, MOG-Ab, MS and HC against selected MOG peptides. All participants showed a positive CD4^+^ T-cell proliferative response with a CDI ≥ 3 to the positive control TTX. We observed no statistically significant differences after challenge with the different MOG peptides between groups (Figures 6, 7A and Table 1). T-cell proliferation with a CDI ≥ 3 for at least one MOG peptide was observed in 40% of AQP4-Ab positive patients, 40% of MOG-Ab positive patients, 63% of MS patients and 50% of HC.

The functional phenotype of proliferating T-cells was characterized by investigating the secretion of the cytokines IL-4, IL-6, IL-17A, GM-CSF, and IFN-ɤ into cell culture supernatants of the CFSE proliferation assay using ELISA. Cytokine concentrations of IL-4 and IL-17A after stimulation with AQP4 peptides, but not after stimulation with TTX, were below the detection limit of ELISA. Quantitative and qualitative values for GM-CSF, IFN-ɤ, or IL-6 levels are shown in Figures 6, 7B–D.

### HLA Association of AQP4-Specific T-Cell Reactivity

**Figure 8:**
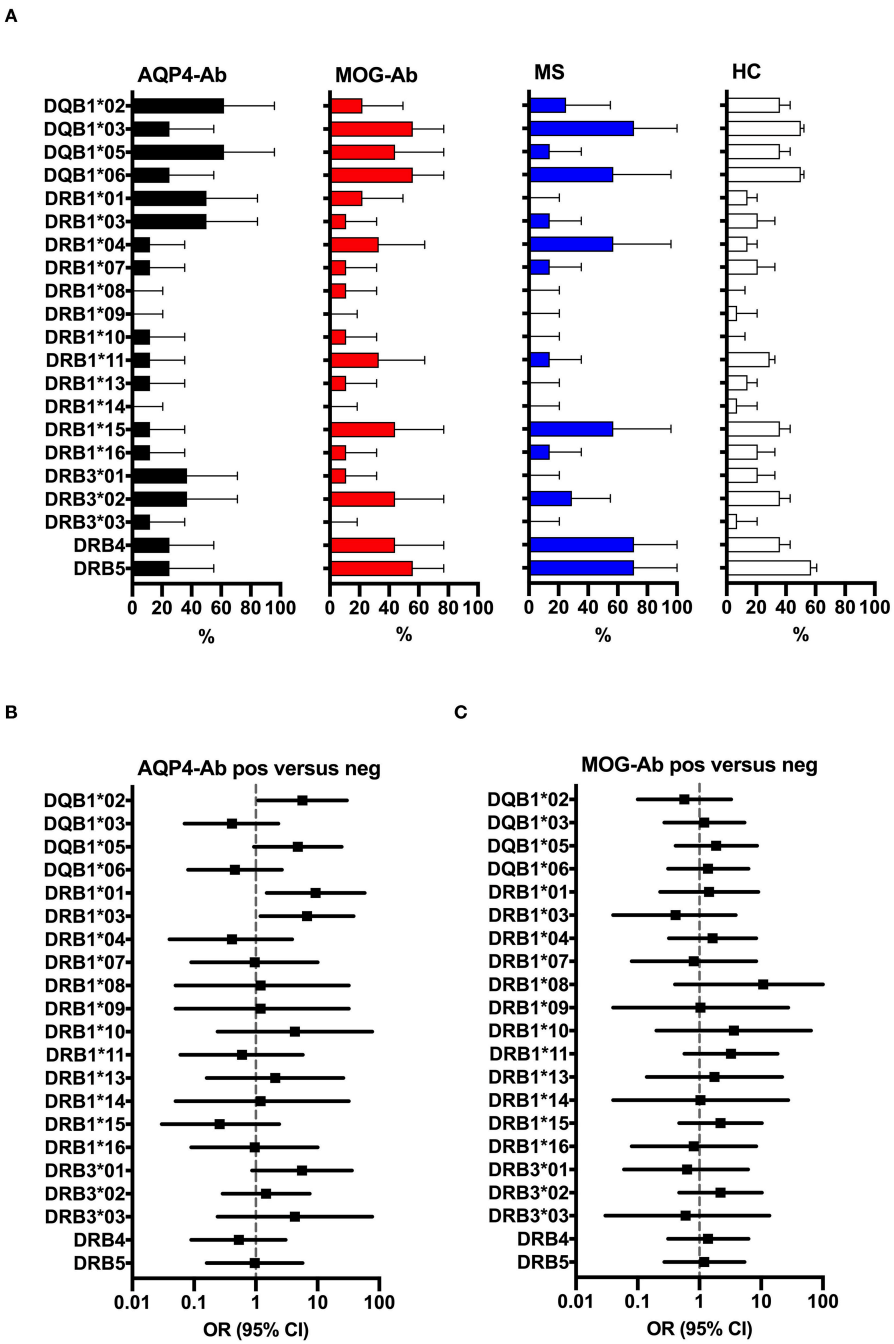
**(A)** HLA allele frequency (in% with 95% confidence intervals indicated by the error bars) of participants with AQP4-Ab (*n* = 8), MOG-Ab (*n* = 9), MS (*n* = 8) and HC (*n* = 14). **(B)** Forest plot displaying Mantel-Haenszel common odds ratio estimates (symbols) with asymptomatic 95% CI (horizontal lines) for the different HLA alleles in participants with AQP4-Ab (*n* = 8) vs. all AQP4-Ab negative participants (*n* = 31). HLA alleles DQB1*02, DRB1*01 and DRB1*03 were overrepresented in participants with AQP4-Ab. **(C)** Forest plot displaying Mantel-Haenszel common odds ratio estimates (symbols) with asymptomatic 95% CI (horizontal lines) for the different HLA alleles in participants with MOG-Ab (*n* = 9) vs. all MOG-Ab negative participants (*n* = 30). AQP4-Ab, aquaporin-4 antibody positive; HC, healthy controls; HLA, human leucocyte antigen; MOG-Ab, myelin oligodendrocyte glycoprotein antibody positive; MS, multiple sclerosis.

Figure 8 shows the overall frequencies of HLA-DQB1, HLA-DRB1 and HLA-DRB3 alleles in participants with AQP4-Ab, MOG-Ab, MS, and healthy controls. The following HLA genotypes were overrepresented in participants with AQP4-Ab (*n* = 8) compared to those without AQP4-Ab (*n* = 31): DQB1^*^02 (OR = 5.71, 95% CI 1.09–30.07), DRB1^*^01 (OR = 9.33, 95% CI 1.50–58.02) and DRB1^*^03 (OR = 6.75, 95% CI 1.19–38.41).

The four study participants reactive with AQP4 p156-170 peptide 156–170 had the following HLA genotypes: Nr. 1 (DQB1^*^02, DQB1^*^05, DRB1^*^01, DRB1^*^03, DRB3^*^01), Nr. 2 (DQB1^*^03, DQB1^*^05, DRB1^*^10, DRB1^*^11, DRB3^*^02), Nr. 3 (DQB1^*^02, DQB1^*^06, DRB1^*^07, DRB1^*^15, DRB4, DRB5), and Nr. 4 (DQB1^*^03, DQB1^*^05, DRB1^*^01, DRB1^*^04, DRB4). However, only HLA DRB1^*^01 was associated with the presence of AQP4 p156-170 reactive T-cells (OR = 31.67, 95% CI 1.30–772.98).

## Discussion

In this study we analyzed peripheral blood T-cell responses to AQP4 and MOG peptides in individuals with AQP4-Ab, MOG-Ab, MS, and HC. We identified significantly increased AQP4-specific CD4^+^ T-cell reactivity to AQP4 peptide 156–170 in 4 of 8 AQP4-Ab positive NMOSD patients, but in none of the other groups. In contrast, we could not detect any significant disease-specific T-cell response to other AQP4 or MOG peptides. AQP4 peptide 156–170 has already been described as a T-cell epitope in NMOSD patients (31) and is also one of the most important B-cell epitopes recognized by AQP4-Ab (14, 15, 58, 59). Three other immunodominant T-cell epitopes/peptides of the AQP4 protein have been described by Varrin-Doyer et al. (31), which were also included in our study. However, we and other authors could not confirm immunodominance for these particular determinants (38, 60). The possible reasons for this discrepancy could be explained by the different methods used (CFSE, ^3^H-thymidine incorporation proliferation assays, cytokine secretion) and the different genetic background, i.e., HLA associations of the study populations. Several studies have identified over-representation of HLA-DPB1^*^0501, HLA-DRB1^*^0301, or HLA-DRB3 in NMO patients (31, 61–63). However, only DRB1^*^0301 but not any of the other HLA alleles was overrepresented in our study population, indicating differences in the genetic background. In contrast, we found an overrepresentation of HLA-DQB1^*^02 and HLA-DRB1^*^01 in our study.

Some AQP4-specific T-cells (particularly against AQP4 peptides 11–30, 61–80, 63–76, 91–110, 139–153, and 281–305) were also present in participants with MOG-Ab, MS, and HC, consistent with previous studies demonstrating antibody response against linear AQP4 peptides (64). The presence of these AQP4-specific T-cell responses may reflect unspecific bystander activation, i.e. T-cell receptor (TCR)-independent activation of autoreactive T-cells by pro-inflammatory cytokines during inflammation, and/or epitope spreading (65).

We found no differences in MOG-specific T-cells between the four different groups with our experimental approach. The reason for the observed results could be explained by ignorance of the immune system of the MOG protein (66). In contrast to the AQP4 protein, which is also highly expressed in the periphery (67, 68) and hence underlies highly regulated mechanisms of self-tolerance (69, 70), MOG is only expressed in the CNS at very low levels (71, 72) and therefore not subject to intense immune surveillance. This might explain why HC showed lower response to AQP4 determinants, but *in vitro* stimulation with MOG peptides also caused profound T cell response in some healthy subjects.

Synthetic MOG peptides used in this study may not accurately represent naturally processed antigen and MHC-presented peptides in an *in vivo* setting (73). One of the major pathogenic mechanisms of MOG-Ab is considered the enhanced presentation of native MOG protein to T cells via Fc receptor mediated internalization of the antigen-Ab complex (74–76). Therefore, it is possible that MOG-reactive T cells can only be detected using intact MOG protein as the antigen. Indeed, Bronge et al. were able to identify increased frequencies of IFN-γ, IL-22 and IL-17A producing MOG-specific T-cells in patients with MS using bead-bound MOG as the antigen (77).

A major limitation of our study is the small number of included participants. This number reflects the expected number for our clinical centers, since NMOSD and MOG-related disorders are rare with a worldwide prevalence of 1–4/100,000 comparatively similar in most populations. Other limitations are that most patients received immunosuppressive therapy during sample collection related to their severe clinical presentations. Most AQP4-Ab positive patients (6/8) and 4/10 MOG-Ab positive patients received immunomodulatory or immunosuppressive treatment at the time of sample collection. Although B-cell depleting therapy is known to affect T-cell responses in patients with MS (78), 3/4 AQP4-Ab patients who had increased T-cell reactivity to AQP4 peptide 156-170 were treated with rituximab and the fourth patient was under high-dose corticosteroids before the initiation of rituximab treatment.

Importantly, the detailed characterization of single peptides, i.e. known “candidate antigens” based on their encephalitogenicity in animal models and/or their immunodominance in humans is crucial for a potential use in antigen-specific tolerance induction therapies (60, 79). For future studies, the implementation of new unbiased approaches may provide additional perspectives. These new strategies differ from previous studies by using combinatorial peptide libraries, which e.g., cover the entire protein or which allow the discovery of novel “unknown” antigens (80). Discrepancies to other studies might be explained by the use of different assays. We and all other investigators in this field face the issue of very low precursor frequencies of CNS antigen-specific T cells in PBMC preparations. However, the CFSE dilution assay used here is a powerful and sensitive method for directly detecting proliferation of rare autoantigen-specific human T-cells (31, 81). Moreover, the late addition of IL-2 during a re-stimulation step (82–84) offers high sensitivity and specificity. This strategy effectively increases the sensitivity for rare antigen-specific T-cells by selectively facilitating the proliferation of T lymphocytes that express the IL-2 receptor alpha-chain CD25 following antigen recognition (82, 85). However, even though our culture conditions promote the survival of mostly proliferating T-cells, other cells might skew the cytokine response. Indeed, it is well acknowledged that the key Th17-polarizing cytokine IL-6 is produced by myeloid cells (monocytes) rather than T-cells. Furthermore, the timing of sample collection might influence the relative abundance of the different cytokines and therefore explain differences to previous studies. Finally, additional factors such as the TCR avidity or the peptide concentration in different assays may play a role for various specificities. The application of (i) a lower peptide concentration (here used 20 μg/ml per peptide is higher compared to other studies), (ii) the use of native protein antigens instead of peptides, (iii) purified memory T-cells instead of PBMC, and/or (iv) the co-culture of T-cells with autologous APC, e.g., monocytes or EBV-transformed B cells, may be critical improvements for future experiments.

To conclude, this report investigates AQP4- and MOG-specific T-cell reactivities in human individuals presenting with AQP4-Ab and MOG-Ab positive demyelinating diseases. Our *in vitro* data corroborates previous findings showing the involvement of AQP4-specific T-cells in AQP4-Ab positive NMOSD and confirms the AQP4 peptide 156-170 as specific T-cell epitope. In contrast, no disease-relevant MOG peptide was identified. Future confirmatory studies using an unbiased approach for epitope discovery in larger cohorts may overcome main limitations of small sample size and the use of a limited collection of synthetic peptides in this study.

## Data Availability Statement

All datasets generated for this study are included in the article/supplementary material.

## Ethics Statement

The studies involving human participants were reviewed and approved by the local Ethics Committee of Medical University of Innsbruck, Austria (study number AN3041) and University of Zurich, Switzerland (KEK ZH 2013-0001) and all patients and controls gave written informed consent. Written informed consent to participate in this study was provided by the participants' legal guardian/next of kin.

## Author Contributions

LH and MRe performed experiments, analyzed and interpreted data, and drafted the manuscript. MRa performed experiments and participated in the analysis and interpretation of the data. VG and MRe designed the study and MRe and AL analyzed and interpreted data and revisited the article critically for important intellectual content. AP performed experiments and analyzed the data. KR, MS, HH, TB, and AL provided patient material and revisited the article critically for important intellectual content. All authors approved the final version of the manuscript.

## Conflict of Interest

The Medical University of Innsbruck and the University Hospital Innsbruck (MRe) receive payments for antibody assays (AQP4 and other anti-neuronal and anti-glial antibodies) and for AQP4 antibody validation assays organized by Euroimmun (Luebeck, Germany). HH has participated in meetings sponsored by, received speaker honoraria or travel funding from Bayer, Biogen, Merck, Novartis, Sanofi-Genzyme, Siemens, Teva, and received honoraria for acting as consultant for Biogen and Teva. KR received speaker honoraria from Merck, Novartis and served as a consultant for PARADIGM-Study, Novartis with no compensation. TB has participated in meetings sponsored by and received honoraria (lectures, advisory boards, consultations) from pharmaceutical companies marketing treatments for multiple sclerosis: Almirall, Bayer, Biogen, Biologix, Bionorica, Genzyme, MedDay, Merck, Novartis, Octapharma, Roche, Sanofi/Genzyme, TG Pharmaceuticals, TEVA-ratiopharm and UCB. His institution has received financial support in the last 12 months by unrestricted research grants (Biogen, Merck, Novartis, Roche, Sanofi/Genzyme) and for participation in clinical trials in multiple sclerosis sponsored by Biogen, Merck, Novartis, Roche, Sanofi/Genzyme, and TEVA. AL received financial compensation and/or travel support for lectures and advice from Biogen, Merck, Novartis, Teva, Genzyme, Bayer, Celgene and he is a co-founder of Cellerys and co-inventor on a patent held by the University of Zurich on the use of peptide-coupled cells for treatment of MS.

The remaining authors declare that the research was conducted in the absence of any commercial or financial relationships that could be construed as a potential conflict of interest.
